# Dust Storms Increase the Risk of Age-Related Macular Degeneration

**DOI:** 10.3390/ijerph19127403

**Published:** 2022-06-16

**Authors:** Chin-Shyan Chen, Conmin Chen, Tsai-Ching Liu

**Affiliations:** 1 Department of Economics, National Taipei University, New Taipei City 23741, Taiwan; stan@mail.ntpu.edu.tw; 2 School of Medicine, Tzu Chi University, Hualien 97004, Taiwan; 107311116@gms.tcu.edu.tw; 3 Department of Public Finance, National Taipei University, New Taipei City 23741, Taiwan

**Keywords:** AMD, dust storm, air pollution, particulate matter, outpatient, inpatient

## Abstract

The main purpose of this study was to examine the association between dust storms (DSs) and age-related macular degeneration (AMD) using a 5 year representative national dataset with one million participants, according to information on DS, meteorology, and air pollution in Taiwan. There were 18,855 AMD outpatient cases and 1080 AMD inpatient cases during 2008–2012. A Poisson time-series model was used for the analysis. The results show that AMD cases are significantly associated with exposure to dust storm events. Average daily numbers of wet and dry AMD outpatient cases increased from 6.03 and 4.26 on no-event days to 8.25 and 6.67, 2 days after DSs. Average daily numbers of wet and dry AMD inpatient cases increased from 0.26 and 0.33 on no-event days to 0.58 and 0.75, 1 day after DSs. Both genders and different age groups are all affected by the occurrence of DSs, especially 1 and 2 days after DS events. Women are at a higher risk of outpatient visits and hospitalizations for relatively severe wet AMD. Although AMD highly correlated with age, this study also found that dry AMD outpatient visits in people under the age of 50 were also found to be significantly associated with DS events. In order to protect the health of eyes and avoid AMD, one should reduce or avoid outdoor activities when DS events occur.

## 1. Introduction

As the global aging population continues to increase, the prevalence of age-related macular degeneration (AMD) has also risen as a consequence. In fact, AMD is the leading cause of visual impairment and loss of vision for the elderly [1]. Wong et al. [2] showed that 8.7% of the population has AMD, and the projected number of people worldwide with AMD will increase from 196 million in 2020 to 288 million in 2040. There are two types of AMD. One is dry (also called nonexudative) AMD, and the other is wet (also called exudative) AMD. Although the latter only accounts for 10–15% of all AMD cases, wet AMD can lead to severe visual impairment and blindness, dramatically affecting an individual’s independence and quality of life [1,3,4,5,6,7]. Moreover, AMD treatments are costly and may not be affordable to everyone in both developed and developing countries, thus potentially imposing a substantial burden on the healthcare sector, society, and global economy. The risk factors of AMD include age, race, smoking, and diet [1], while hypertension and diabetes are found to potentially relate to AMD development [1,6].

Another important factor that affects AMD is ambient air pollution. Exposure to air pollution is associated with AMD [8]. Ambient air pollution, such as fine particulate matter (PM_2.5_), increases the risk of AMD [9], while long-term exposures to PM_10_, NO_2_, and CO may raise its prevalence [10]. Since air pollution can induce oxidative stress and inflammation, it is likely to escalate the incidence of AMD [8,9], because the retina is one of the most oxygen-consuming tissues in the human body due to the need for metabolism. When the accumulation of reactive oxygen species is unbalanced, it can lead to oxidative stress; thus, air pollution causes oxidative stress and consequent inflammation [9,10].

Ambient air pollution can come from cars, factories, forest fires, and dust storms (DSs). DSs often arise in the Middle East, Central Asia, North America, Central Africa, and Australia. In Taiwan, DSs originating from the deserts of Mongolia and China, can affect the western part of the island during the winter and spring [11]. The pollutants in the dust combined with intensified desertification in China are resulting in more and longer DS events [12,13]. Particulate matter (PM) concentration levels also increase due to DS [13,14,15,16,17].

Past studies have identified associations between DSs and hospitalization due to respiratory and cardiovascular diseases [11,12,13,14,15,16,18,19,20]. However, the effects of DSs on the eye, which is directly exposed, are rarely discussed. Only two studies showed that DSs impact a person’s eyes. Chien et al. [21] reveal significantly acute impacts on children’s conjunctivitis clinic visits during DS periods, especially school children. Mu et al. [22] indicated that the occurrence of eye lacrimation is related to DSs. To the best of our knowledge, there is no study analyzing the relationships between DSs and AMD. Using a 5 year population-based and representative national dataset, the main purpose of this study was to fill the gap in the literature by examining the association of DS events with the daily number of AMD hospital admissions and outpatient visits in Taiwan.

## 2. Materials and Methods

### 2.1. Data

AMD outpatient visits and hospitalizations during 2008–2012 were retrieved from a representative sample of longitudinal health insurance data with one million cases built upon the population-based National Health Insurance Research Database (NHIRD) in Taiwan. AMD can be divided into dry AMD and wet AMD. Dry AMD is a chronic atrophic eye disease that can lead to blindness after decades. This type accounts for about 85–90% of overall cases. The diagnosis codes (ICD-9-CM) are 362.51 and 362.57. Wet AMD is a neovascular disease that may cause vision loss over a period of weeks to months and requires urgent treatment. This type accounts for about 10–15% of overall cases. The diagnostic codes (ICD-9-CM) are 362.52 and 362.53 [23,24].

Air pollution data were taken from Taiwan’s Environmental Protection Administration (TEPA) database. TEPA defines DS events using the PM_10_ concentrations measured by five indicator observation stations, including Yilan, Wanli, Guanyin, Matsu, and Yang Ming. DSs in Taiwan mainly come from overseas areas, and the locations of these indicator observation stations are all on offshore islands or in northern coastal areas. Therefore, the measured concentrations are not easily affected by local stationary sources of air pollution and mobile sources of air pollution. Normally, the 24 h moving average concentration of PM_10_ is about 50 μg·m^−3^, but PM_10_ can rise to above 100 μg·m^−3^ when DSs emanate from overseas. TEPA uses 100 μg·m^−3^ as the criterion (a total of 11 DS events occurred between 2008 and 2012) for measurement.

The main substances of air pollution include PM_10_, PM_2.5_, CO, O_3_, NO_2_, and SO_2_. When DSs occur, both PM_10_ and PM_2.5_ increase simultaneously, because the concentrations of these particles are included in the DS data. Therefore, this study used other air pollutants to represent the impact of air pollution. According to the monitoring data of TEPA, only the concentrations of PM_10_, PM_2.5_, and O_3_ reached unhealthy levels in recent years, while the other substances were at good levels; hence, this paper used O_3_ as an explanatory variable.

According to previous studies, the concentration of NO_2_ has an important impact on AMD; thus, it was also included in the research model [8,9,10]. The concentrations of O_3_ and NO_2_ were calculated from daily averages of 60 air quality observation stations across Taiwan. Weather data were taken from Taiwan’s Central Weather Bureau database. The ambient temperature was calculated from the daily average temperature of 23 meteorological observation stations across Taiwan.

### 2.2. Methods

This study employed the Poisson time-series analysis proposed by Brännäs [25,26]. The main reason is that the data of daily outpatient visits and hospitalizations are aggregated time series of count data. We separated the samples by disease type and severity. AMD was divided into dry AMD and wet AMD, as well as inpatient AMD and outpatient AMD. In addition, according to past studies, gender and age are also important factors affecting AMD [27,28]. Therefore, samples were stratified according to gender (male and female) and age groups (below 50, 51–60, 61–70, 71–80, and over 81). The empirical model is defined below.

(1) $$AMD_{t}^{\;}=\alpha_{0}^{\;}+\alpha_{1}^{\;}t_{\;}^{}+\sum_{i=0}^{3}\;\alpha_{2i}^{\;}Day_{i}^{\;}+\sum_{j=1}^{p}\;\alpha_{3j}^{\;}AMD_{t-j}^{\;}+\alpha_{4}^{\;}X_{t}^{\;}+\varepsilon_{t}.$$

The dependent variable, **AMD_t_**, is the daily number of AMD outpatient visits or hospitalizations; **α_0_** is the intercept term; **t** is the time trend; **Day_i_** is a dummy variable for DS events. The impact of DSs on AMD mainly occurs through particulate matter (PM). Particulate matter enters the lungs from the respiratory tract and then enters the blood circulation from the pulmonary blood vessels. Particulate matter flowing through the eyes can lead to AMD. This process can take days to have an effect; therefore, we used Day_0_ to capture the immediate effects and Day_1_ to Day_3_ to capture the delayed effects (with no-DS days as the reference group). **AMD_t−j_** is the autoregressive term of AMD. We selected the optimal autoregressive lags on the basis of the Ljung–Box test and autocorrelation function (ACF) in order to minimize the autocorrelation in error. **X_t_** represents other confounders, including atmospheric data such as ambient temperature, NO_2_, O_3_, and season (with summer as the reference group). Lastly, **ε_t_** is the error term.

## 3. Results

### 3.1. Descriptive Statistics

#### 3.1.1. Outpatient Care

Table 1 lists the numbers of daily AMD outpatient cases according to days after DS outbreaks. There were 18,855 AMD outpatient cases in total with 11,043 wet AMD and 7812 dry AMD cases during 2008–2012. The cases of wet AMD outpatient care exceeded those of dry AMD.

**Figure 1:**
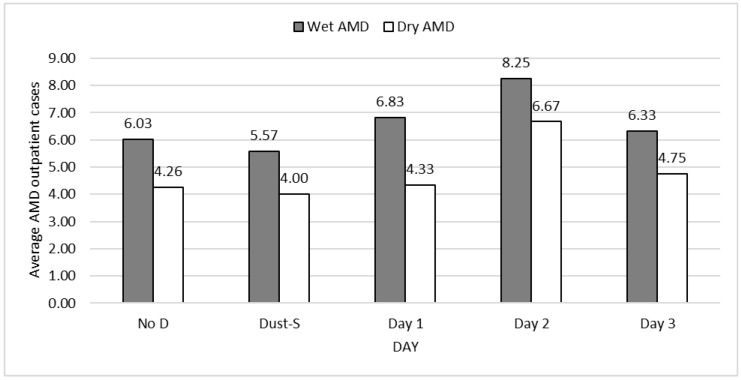
Average daily AMD outpatient visits according to days after dust storms, Taiwan, 2008–2012.

Figure 1 depicts the trend in outpatient visits of AMD during DS events. Average daily numbers of both wet and dry AMD outpatient cases exhibited an increased tendency with a peak on day 2 after DSs, suggesting that DSs resulted in more AMD outpatient visits on days 1 and 2 after DSs compared to the no-event day, regardless of type (6.83 and 8.25 vs. 6.03; 4.33 and 6.67 vs. 4.26). The average number of wet AMD cases ranged from 5.57 to 8.25, which is far more than that of dry AMD cases (4.00–6.67), implying that the occurrence of wet AMD outpatient visits was approximately 30–60% higher than that of dry AMD visits.

Table 1 also shows the trend of AMD outpatient visits during DS events according to gender and age. Among a total of 11,043 wet AMD outpatient cases, around 60% (*n* = 6567) were males. The average numbers of wet AMD outpatient cases increased from 3.59 on no-event days to 5.08, 2 days after DSs for males and increased from 2.44 on no-event days to 3.33, 1 day after DSs for females. Males appeared to have a higher risk than females. DSs seemed to impact the daily numbers of wet AMD outpatient cases regardless of gender. Among 7812 dry AMD outpatient cases, 4587 cases (58.7%) were attributed to males, much higher than female cases at 3225 (41.3%). The average daily numbers of dry AMD outpatient cases both increased from 2.50 and 1.76 on no-event days to 4.0 and 2.67, 2 days after DS for males and females, respectively. This suggests that there also was an association between DSs and dry AMD outpatient care uses.

The age group of 71–80 had the largest number of wet AMD outpatient cases (*n* = 2933), constituting about 30%, while the age group of ≤50 had the lowest number (*n* = 1046). Age groups of ≤50, 61–70, and 71–80 appeared to see increased wet AMD outpatient cases from 0.57, 1.49, and 1.59 on no-event days to 0.67, 1.92, and 2.50, 2 days after DS outbreaks, while age groups of 51–60 and ≥81 increased from 1.04 and 1.33 on no-event days to 1.58 and 2.17, 3 days and 1 day after DS onset, respectively. Similar to wet AMD, the number of daily dry AMD outpatient cases increased with advanced age, from 287 cases (3.7%) for ages ≤50 to 2798 cases (35.8%) for ages ≥81. The age groups of ≤50, 51–60, 71–80, and ≥81 saw more dry AMD outpatient cases, ranging from 0.15, 0.36, 1.42, and 1.53 on no-event days to 0.42, 0.75, 2.50, and 2.17, 2 days after DSs, while cases in the age group of 61–70 increased from 0.79 to 1.50, 3 days after DS. The data indicate that DS occurrences correlated with the risk of both wet and dry AMD outpatient care use, regardless of age.

#### 3.1.2. Inpatient Care

There were a total of 1080 AMD inpatient cases according to days after DS outbreaks, as shown in Table 2, with 471 for wet AMD and 609 for dry AMD, respectively. In contrast to outpatient care, the cases of wet AMD inpatient care appeared to be lower than those for dry AMD inpatient care.

**Figure 2:**
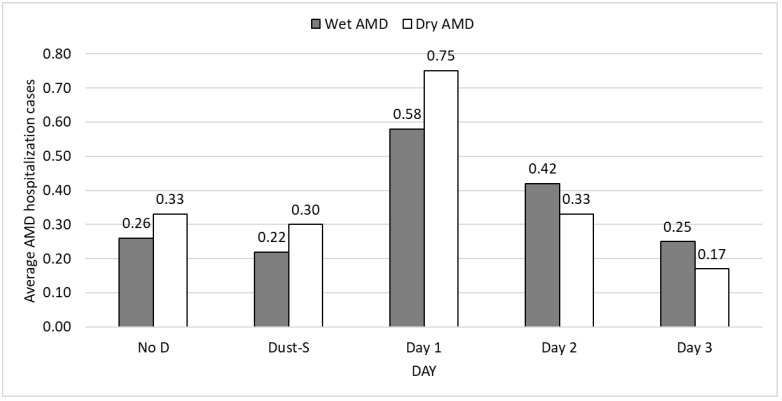
Average daily AMD inpatients according to days after dust storms, Taiwan, 2008–2012.

Figure 2 depicts the trend in hospitalizations of AMD during DS events. Average daily numbers of both wet and dry AMD inpatient cases exhibited an increasing trend with a peak on 1 day after DSs, suggesting that DSs resulted in more AMD hospitalizations 1 day than on no-event days, regardless of type (0.58 vs. 0.26; 0.75 vs. 0.33). This suggests that DSs led to more AMD hospital admissions on days after DSs than that on no-event days, whether wet AMD or day AMD. However, unlike outpatient visits, the average numbers of wet AMD hospital admissions (0.22–0.58) were lower than those of dry AMD hospital admissions (0.30–0.75) before 1 day after DSs, implying that the occurrences of dry AMD inpatient care were approximately 30% higher than those of wet AMD inpatient care. However, the average numbers of wet AMD hospital admissions (0.42 and 0.25) were higher than those of dry AMD hospital admissions (0.33 and 0.17), 2 and 3 days after DSs.

Table 2 shows the trend of AMD inpatient care during DS events according to gender and age. Among 471 wet AMD inpatient cases, about 60% were attributed to males (*n* = 279) with the ratio between males and females at 1.45:1.00. The average number of daily wet AMD inpatient cases on no-event days for males and females was 0.15 and 0.10, respectively. For males, the number of wet AMD inpatient cases increased to 0.33, 1 day after DSs (two times higher compared to no-event days). A considerable change was also observed for females before and after DS. The average numbers of daily AMD inpatient cases on no-dust days, dust event days, 1 day after DSs, and 2 days after DSs were 0.10, 0.17, 0.25, and 0.33, respectively. This indicates that wet AMD inpatient care increased with DS outbreaks for females. This study indicated a total of 609 dry AMD inpatient cases, where 399 cases were males (65.5%) and 210 were females (34.5%). For both males and females, the average number of dry AMD inpatient cases was double 1 day after DS onset, going from 0.22 and 0.11 to 0.50 and 0.25. This indicates that the risk of dry AMD hospital admission on day 1 after DSs was almost twofold greater than that on no-event days, regardless of gender.

More than one-third of wet AMD inpatient cases were in the age group 61–70 (*n* = 152), while only one-tenth of cases were in patients aged under 50 (*n* = 50). Age groups of 51–60, 71–80, and ≥81 appeared to see more wet AMD inpatient cases, ranging from 0.05, 0.06, and 0.04 on no-event days to 0.25, 0.17, and 0.08, 1 day after DS outbreaks, while cases in age groups of 61–70 and ≤50 increased from 0.08 and 0.03 on no-event days to 0.17 and 0.08, 2 days after DS onset. This implies that the average number of inpatient cases of wet AMD 1 or 2 days after DS events was 2–5 times greater than that of cases on no-event days for various age groups. The numbers of daily dry AMD inpatient cases increased with advanced age, ranging from seven cases (1.1%) for age ≤50 to 271 cases (44.5%) for age ≥81. For the three age groups of 61–70, 71–80, and ≥81, the average numbers of dry AMD inpatient cases 1 day after events all reached a peak, going from 0.05, 0.12, and 0.15 on no-event days to 0.08, 0.25, and 0.42, respectively. This suggests that the elderly aged beyond 61 had at least 1.5 times higher risk of having a dry AMD hospital admission 1 day after DS outbreaks. Among the younger age group of ≤50, the largest number of cases occurred on the event day, rising from 0.00 on no-event days to 0.04. However, there was no difference before and after DS for the age group of 51–60.

### 3.2. Regression Results

#### 3.2.1. Outpatient Care

Table 3 shows the Poisson autoregressive time-series analysis between wet AMD outpatient visits and DS events. After controlling for temperature, NO_2_, O_3_, trends, and seasonal factors, the results indicated that the day of DS did not have a significant impact on wet AMD outpatient visits. However, compared to no-event days, there were significantly higher numbers of outpatient visits for the female group 1 day after DSs. This indicates that the risk of wet AMD outpatient care 1 day after DS onset was higher than that on days without DSs among females, while no disparities were observed for males. Regarding the five age groups, only the elderly group (age ≥81) appeared to have significantly higher numbers of AMD outpatient visits 1 day after DSs. This indicates that DS events had a specific impact on those aged above 80. Moreover, the positive coefficients of the trend variable for both males and females and most age groups indicate that the risk of wet AMD outpatient care increased over time. Wet outpatient AMD visits increased significantly with a rise in temperature for the total population, male, and age ≥81 groups, as well as with a rise in the amount of NO_2_ for the total population, female, age 61–70, and age 71–80 groups.

Table 4 lists the regression results between daily dry AMD outpatient visits and DS events. Significantly higher numbers of dry AMD outpatient visits were not found on DS days compared to no-DS days. However, the numbers of dry AMD outpatient visits significantly spiked 2 days after DSs for the total population. One of the four event dummies, 2 days post DS, also presented a positive coefficient for the subgroups of age ≤50 and age 71–80. This finding suggests that patients of age below 50 or age 71–80 had a higher risk of dry AMD outpatient care 2 days after DS outbreaks compared to no-event days. Furthermore, the trend variable indicated that dry AMD outpatient visits increased significantly over time. However, the impact was mixed for different age groups. Dry outpatient AMD visits increased significantly with a rise in temperature for the total population, male, female, age 71–80, and age ≥ 81 groups, as well as with a rise in the amount of NO_2_ for most subgroups except for male, age ≤ 50, and age ≥ 80 groups.

#### 3.2.2. Inpatient Care

Table 5 shows the association between daily wet AMD inpatient care use and DS events. We note that, among the four DS dummies, only one, 2 days post DS, was statistically significant with a positive coefficient for females, but none of the four event dummies were shown to have a significant impact for males. This indicates that wet AMD hospital admissions were increased 2 days after DSs for the female subgroup compared to days without DSs. Among the five age subgroups, only one of the four DS dummies, 1 day post DS, had a statistically significant and positive coefficient for the subgroup of age 51–60. This result implies that DS had a delayed effect on the number of daily wet AMD hospital admissions for the specific age group of 51–60. Moreover, the number of wet AMD hospital admissions increased significantly with temperature for the total population, male, age 51–60, and age ≥81 groups. The trend variable showed that only the elderly age beyond 80 tended to have more daily wet AMD inpatient cases over time.

Table 6 lists the association between daily dry AMD hospitalizations and DS events. The results show that one of the four event variables, 1 day post DS, had a statistically significant and positive coefficient in the total population and male group, indicating that the risk of dry AMD inpatient care use was significantly higher 1 day after DS events compared to no-event days for males and the total population. When the estimation was stratified by age groups, the positive and significant coefficient 1 day post DS was also present for the elderly age group ≥81, implying that the risk of dry AMD hospital admissions tended to be higher 1 day after DS outbreaks for those aged beyond 80 compared to days without DS. Unlike the estimation results of AMD outpatient visits and wet AMD inpatient care, the negative coefficient of the trend variable suggested that the number of daily dry AMD hospitalizations decreased significantly over time. A rise in the amount of NO_2_ tended to significantly increase daily dry AMD hospitalizations.

## 4. Discussion

Air pollution is associated with respiratory and cardiovascular diseases such as stroke, asthma, and pneumonia [11,12,13,14,15,16,29]. Since eyes are directly exposed to air pollutants, it is likely that air pollution can cause eye-related diseases. However, only a few studies focused on examining the impact of air pollution on eye diseases [10,30]. One of the major sources of air pollution comes from DSs, but research on the correlation between DSs and eye diseases is rare. There are only two studies that explored the impact of DSs on conjunctivitis and eye lacrimation [21,22]. AMD is one of the leading eye diseases that causes blindness in the elderly [1]. However, no examination has been conducted for the association between DSs and AMD. Therefore, this study examined the associations between DS events and AMD healthcare using a large national health insurance research database from 2008 to 2012 in Taiwan.

The present study observed that AMD outpatient visits and hospitalizations were associated with DS events, yet the effect was not immediate, but delayed. Our results indicated that DS event days did not result in a significant higher number of AMD outpatient and inpatient cases. However, a significantly higher number of AMD cases were observed 1 and 2 days after DS events. Since DSs can increase PM concentration levels, the delayed effect may be explained by the delayed biological effects following exposure to residual PM after DS events [12,29]. An increase in PM concentration levels initiates systemic oxidative stress and consequent lipid peroxidation, which activates the innate immune system and increases inflammation in the retina and cells, potentially resulting in increased risk of AMD [10]. This process takes some days. Similar delayed effects can also be found in the literature regarding the impact of DS events on other diseases [11,12,13,19]. Consistent with previous studies that examined the relationships between ambient air pollution and AMD [8,9,10], this study confirmed a positive relationship between DSs and AMD. Our results are also consistent with Chien et al. [21] and Mu et al. [22], who showed that the prevalence of eye diseases increased after DS events.

On the basis of the total population, we compared the difference between wet AMD and dry AMD results and found that only dry (but not wet) AMD outpatient and inpatient numbers were significantly higher 1 and 2 days after DSs. However, when the data were stratified by gender, we found that wet outpatient visits and hospitalizations 1 and 2 days after DSs had a significantly higher number than no-event days for females. A significantly higher number of dry AMD cases and hospitalizations was found 1 day after DSs for males only. In other words, women were more likely to undergo more serious wet AMD outpatient and inpatient care than men following DS events. Some previous studies indicated a relatively higher prevalence of wet AMD in women. Rudnicka et al. [31] showed a higher risk (OR = 1.2) of wet AMD in women compared with men. A study conducted in southwestern Taiwan by Huang et al. [32] also noted that the prevalence of early AMD was higher in men than in women.

When stratifying the data into different age groups, the results indicated that the impact of DS events on AMD across all age groups was comprehensive. AMD is the leading cause of irreversible blindness in people above age 50 years old [33,34], and age is by far the most significant factor for AMD [1]. The prevalence of AMD is gradually increasing as a consequence of exponential population aging [2]. It is not surprising to find that numbers of both wet AMD outpatient and dry AMD inpatient visits were significantly higher 1 day after DSs than on no-event days for the groups over 81 years old. People aged 71–80 and 51–60 also tended to have a significantly more dry AMD outpatient visits 2 days after DSs and wet AMD hospitalizations 1 day after DSs, respectively. Surprisingly, a positive and significant association between dry AMD outpatient visits 2 days after DSs appeared among people under 50 years old. Even though dry AMD is the most common type of AMD, incurring almost no vision loss, it can progress to wet AMD [4,5,6]. However, a younger age of getting dry AMD results in a sooner transition into wet AMD and a subsequent risk of visual impairment. People under 50 still have to be careful about DSs and AMD, despite the literature suggesting that AMD is more common in people 50 years of age or older.

Our results also showed a significantly positive association between temperature and pollutants and AMD healthcare. High temperatures tend to cause more dry and wet AMD care, especially for males and the elderly. High amounts of NO_2_ and O_3_ are also likely to cause increased dry and wet AMD cases. Lastly, the overall trend in AMD outpatient care increased, except for the <50 and 71–80 age groups in dry AMD outpatient care. A growing trend in wet AMD hospitalizations was also found in the ≥81 age group. Conversely, dry AMD hospitalizations trended downward regardless of gender and age group.

The strengths of this research include the use of a large sample size and highly accurate AMD medical data from the NHI research database with information on DSs, meteorology, and air pollution. Our findings provide evidence that the association between AMD and DS does exist and add to the growing evidence of the damaging effects of ambient air pollution on AMD. However, some limitations existed in our study. First, personal exposure levels to DS events were unknown. We used NHI data to identify patients with AMD healthcare use without knowing if this was because of DS events. Second, our AMD data were aggregated daily medical data. Personal risk factors for AMD such as smoking, BMI, and hypertension, therefore, could not be controlled for in the study. Third, despite the increase in daily AMD cases following DS, it should be remembered that the total number of AMD cases associated with DS were still much lower than the overall AMD cases, as DSs only occurred 11 times over a 5 year period.

## 5. Conclusions

This is the first study to show that AMD cases are significantly associated with exposure to dust storm events. Both genders and different age groups were all affected by the occurrence of DSs, especially 1 and 2 days after DS events. Women were at a higher risk of utilization of outpatient visits and hospitalizations for relatively severe wet AMD. Although AMD highly correlated with age, this study also found that dry AMD outpatient visits in people under the age of 50 were significantly associated with DS events. In other words, younger people cannot ignore the impact of DS events on AMD. In order to protect the eyes and avoid AMD, one should reduce or avoid outdoor activities during DS events. The government should also use the media to provide early information on DS events. The findings of this study help to further understand the relationship between eye health and ambient air pollution.

## Figures and Tables

**Table 1:** Average daily AMD outpatient visits according to days after DSs stratified by gender and age.

	No	Day 0	Day 1	Day 2	Day 3	Obs.
Wet AMD						
Total	6.03	5.57	6.83	8.25	6.33	11,043
Male	3.59	3.35	3.50	5.08	3.50	6567
Female	2.44	2.22	3.33	3.17	2.83	4476
≤50	0.57	0.61	0.33	0.67	0.50	1046
51–60	1.04	0.96	1.33	1.42	1.58	1921
61–70	1.49	1.22	1.25	1.92	1.33	2714
71–80	1.59	1.57	1.75	2.50	2.25	2933
≥81	1.33	1.22	2.17	1.75	0.67	2429
Dry AMD						
Total	4.26	4.00	4.33	6.67	4.75	7812
Male	2.50	2.43	2.33	4.00	3.17	4587
Female	1.76	1.57	2.00	2.67	1.58	3225
≤50	0.15	0.13	0.25	0.42	0.17	287
51–60	0.36	0.30	0.33	0.75	0.17	662
61–70	0.79	0.52	0.67	0.83	1.50	1447
71–80	1.42	1.70	1.25	2.50	1.25	2618
≥81	1.53	1.35	1.83	2.17	1.67	2798

**Table 2:** Average daily AMD inpatient care according to days after DSs stratified by gender and age.

	No	Day 0	Day 1	Day 2	Day 3	Obs.
Wet AMD						
Total	0.26	0.22	0.58	0.42	0.25	471
Male	0.15	0.04	0.33	0.08	0.25	279
Female	0.10	0.17	0.25	0.33	0.00	192
≤50	0.03	0.00	0.00	0.08	0.00	50
51–60	0.05	0.00	0.25	0.08	0.08	92
61–70	0.08	0.17	0.08	0.17	0.00	152
71–80	0.06	0.04	0.17	0.08	0.08	107
≥81	0.04	0.00	0.08	0.00	0.08	70
Dry AMD						
Total	0.33	0.30	0.75	0.33	0.17	609
Male	0.22	0.22	0.50	0.25	0.08	399
Female	0.11	0.09	0.25	0.08	0.08	210
≤50	0.00	0.04	0.00	0.00	0.00	7
51–60	0.01	0.00	0.00	0.00	0.00	22
61–70	0.05	0.04	0.08	0.00	0.08	83
71–80	0.12	0.09	0.25	0.17	0.00	226
≥81	0.15	0.13	0.42	0.17	0.08	271

**Table 3:** Poisson autoregressive analysis for wet AMD outpatient care.

	Total	Male	Female	≤50	51–60	61–70	71–80	≥81
Intercept	0.0980	−0.0982	−1.2900 ***	−0.3380	−2.0440 ***	−1.5910 ***	−1.0490 **	−1.8150 ***
	(0.1720)	(0.2140)	(0.2560)	(0.5130)	(0.3760)	(0.3050)	(0.3270)	(0.3460)
Day of DS	0.0650	0.0968	0.0132	0.1220	0.0515	−0.0408	0.2130	−0.0027
	(0.0971)	(0.1200)	(0.1460)	(0.2760)	(0.2140)	(0.1820)	(0.1810)	(0.1940)
1 day post DS	0.1820	0.0144	0.3770 *	−0.4520	0.2790	−0.2330	0.2470	0.5290 *
	(0.1260)	(0.1690)	(0.1720)	(0.5340)	(0.2600)	(0.2550)	(0.2500)	(0.2120)
2 days post DS	0.1600	0.1730	0.1120	0.2450	0.1260	0.0592	0.3260	0.0821
	(0.1140)	(0.1410)	(0.1730)	(0.378)	(0.2520)	(0.2060)	(0.2040)	(0.2280)
3 days post DS	−0.0607	−0.1350	0.0256	−0.2210	0.3360	−0.2600	0.2750	−0.6990
	(0.1300)	(0.1700)	(0.1830)	(0.4280)	(0.2400)	(0.2460)	(0.2160)	(0.3680)
Temperature	0.0096 *	0.0136 **	0.0036	0.0020	0.0070	0.0075	0.0087	0.0162 *
	(0.0038)	(0.0048)	(0.0057)	(0.0115)	(0.0083)	(0.0067)	(0.0073)	(0.0076)
NO_2_	0.0113 ***	0.0076	0.0155 **	0.0007	0.0140	0.0145 *	0.0135 *	0.0081
	(0.0033)	(0.0041)	(0.0049)	(0.0100)	(0.0071)	(0.0058)	(0.0063)	(0.0066)
O_3_	−0.0004	−0.0006	0.0008	−0.0050	−0.0023	0.0012	0.0043	−0.0037
	(0.0013)	(0.0016)	(0.0020)	(0.0041)	(0.0029)	(0.0023)	(0.0025)	(0.0026)
Trend	0.0104 ***	0.0081 ***	0.0137 ***	0.0017	0.0134 ***	0.0116 ***	0.0081 ***	0.0132 ***
	(0.0006)	(0.0008)	(0.0009)	(0.0019)	(0.0014)	(0.0011)	(0.0012)	(0.0012)
Spring	−0.0602	−0.0549	−0.0633	0.0299	0.0009	−0.0756	−0.2730 ***	0.0843
	(0.0414)	(0.0514)	(0.0624)	(0.1260)	(0.0911)	(0.0721)	(0.0816)	(0.0810)
Autumn	0.0035	0.0006	−0.0019	0.0898	0.0551	−0.1260 *	−0.0074	0.0528
	(0.0341)	(0.0421)	(0.0515)	(0.1030)	(0.0754)	(0.0604)	(0.0649)	(0.0681)
Winter	−0.0808	−0.0611	−0.1010	−0.0352	−0.0476	−0.2060 *	−0.1060	0.0154
	(0.0530)	(0.0660)	(0.0792)	(0.1610)	(0.1160)	(0.0932)	(0.1020)	(0.1060)
AR1	0.0086	0.0060	−0.0016	0.0003	0.0060	0.0365	0.0079 *	−0.0007
AR2	0.0043	0.0039	−0.0035	−0.0021	−0.0072	−0.0137	0.0024	0.0010
AR3	0.0035	0.0052	−0.0025	0.0155 *	−0.0036	0.0104	−0.0001	0.0003
AR4	0.0049	0.0034	−0.0011	0.0036	−0.0111	0.0073	−0.0022	−0.0024
AR5	0.0019	0.0006	0.0050	−0.0048	−0.0010	−0.0016	0.0055	0.0004
AR6	−0.0056	−0.0056	−0.0066	0.0022	−0.0080	−0.0242	−0.0089	0.0091
AR7	−0.0026	0.0009	0.0748 **	0.0700 **	0.0712 **	0.0125	0.0250	−0.0218
*N*	1820	1820	1820	1820	1820	1820	1820	1820

* *p* < 0.05, ** *p* < 0.01, *** *p* < 0.001. Reference group: no-event summer day. The notation AR(k) indicates an autoregressive model of order k.

**Table 4:** Poisson autoregressive analysis for dry AMD outpatient care.

	Total	Male	Female	≤50	51–60	61–70	71–80	≥81
Intercept	0.6720 ***	0.4490	−0.6580 *	0.0724	−3.2920 ***	−1.3640 **	0.5820	−1.1040 ***
	(0.1960)	(0.2530)	(0.2960)	(0.9480)	(0.6320)	(0.4300)	(0.3250)	(0.3190)
Day of DS	−0.0208	0.0139	−0.0646	−0.1810	−0.0334	−0.3740	0.1590	−0.0368
	(0.1110)	(0.1410)	(0.1710)	(0.5820)	(0.3760)	(0.2890)	(0.1650)	(0.1840)
1 day post DS	−0.0036	−0.0807	0.0970	0.4870	0.0773	−0.1490	−0.1770	0.1250
	(0.1540)	(0.2090)	(0.2180)	(0.6230)	(0.5160)	(0.3680)	(0.2800)	(0.2260)
2 days post DS	0.2730 *	0.3040	0.2340	0.9640 *	0.3950	−0.1400	0.4140 *	0.1880
	(0.1230)	(0.1580)	(0.1880)	(0.4870)	(0.3420)	(0.3250)	(0.1960)	(0.2070)
3 days post DS	−0.0405	0.0743	−0.2520	0.1250	−0.8230	0.4100	−0.1910	−0.0750
	(0.1450)	(0.1760)	(0.2440)	(0.7500)	(0.7320)	(0.2490)	(0.2730)	(0.2330)
Temperature	0.0161 ***	0.0167 **	0.0154 *	0.0023	0.0039	0.0183	0.0202 **	0.0153 *
	(0.0044)	(0.0057)	(0.0066)	(0.0215)	(0.0139)	(0.0096)	(0.0073)	(0.0071)
NO_2_	0.0153 ***	0.0092	0.0217 ***	0.0244	0.0337 **	0.0188 *	0.0171 **	0.0077
	(0.0038)	(0.0049)	(0.0056)	(0.0183)	(0.0120)	(0.0082)	(0.0063)	(0.0062)
O_3_	0.0013	0.0035	−0.0016	−0.0040	0.0113 *	−0.0001	0.0005	0.0009
	(0.0015)	(0.0019)	(0.0023)	(0.0076)	(0.0047)	(0.0033)	(0.0025)	(0.0024)
Trend	0.0029 ***	0.0014	0.0052 ***	−0.0115 **	0.0085 ***	0.0049 **	−0.0047 ***	0.0093 ***
	(0.0007)	(0.0009)	(0.0011)	(0.0035)	(0.0023)	(0.0016)	(0.0012)	(0.0011)
Spring	0.0422	0.0179	0.0783	−0.1580	−0.0862	0.0666	0.0678	0.0225
	(0.0480)	(0.0616)	(0.0720)	(0.2370)	(0.1610)	(0.1050)	(0.0785)	(0.0760)
Autumn	0.0699	0.0153	0.1380 *	−0.1370	0.2720 *	0.0245	0.0666	0.0450
	(0.0397)	(0.0509)	(0.0598)	(0.1980)	(0.1300)	(0.0882)	(0.0653)	(0.0629)
Winter	−0.0174	−0.0006	−0.0404	−0.2570	−0.1300	0.0180	−0.0209	−0.0011
	(0.0614)	(0.0794)	(0.0919)	(0.3020)	(0.1990)	(0.1340)	(0.1020)	(0.0990)
AR1	0.0081	0.0047	0.0052	−0.0082	−0.0001	0.0000	0.0062 *	−0.0018
AR2	0.0128 *	0.0148 ***	0.0014	−0.0069	0.0060	0.0043	0.0033	0.0010
AR3	0.0057	0.0150 **	0.0024	−0.0062	−0.0077	−0.0046	−0.0015	0.0033
AR4	−0.0025	0.0035	0.0044	−0.0059	−0.0089	0.0115	0.0020	0.0008
AR5	0.0102	0.0034	0.0048	−0.0046	−0.0086	0.0012	0.0050	0.0002
AR6	0.0061	−0.0054	0.0000	−0.0098	−0.0070	−0.0104	0.0101	−0.0041
AR7	−0.0035	−0.0216	0.0176	0.0105	0.0201	−0.0160	0.0018	−0.0560 *
*N*	1820	1820	1820	1820	1820	1820	1820	1820

* *p* < 0.05, ** *p* < 0.01, *** *p* < 0.001. Reference group: no-event summer day. The notation AR(k) indicates an autoregressive model of order k.

**Table 5:** Poisson autoregressive analysis for wet AMD inpatient care.

	Total	Male	Female	≤50	51–60	61–70	71–80	≥81
Intercept	−1.5410 *	−2.9960 **	−1.1430	−3.6810	−4.0970 *	−0.2970	−1.1780	−11.8800 ***
	(0.7840)	(1.0290)	(1.1930)	(2.3310)	(1.7960)	(1.4270)	(1.5650)	(2.3090)
Day of DS	−0.0969	−1.3500	0.7020	−14.6200	−13.4300	0.8670	−0.0693	−14.3300
	(0.4610)	(1.0090)	(0.5200)	(1140.4000)	(484.7000)	(0.5500)	(1.0420)	(938.7000)
1 day post DS	0.8020	0.9300	0.6280	−15.0400	1.8500 **	−0.1810	1.2130	0.8350
	(0.4110)	(0.5420)	(0.6170)	(1716.9000)	(0.6660)	(1.0830)	(0.8180)	(1.0340)
2 days post DS	0.4480	−0.5850	1.0930 *	0.9840	0.5550	0.7260	0.2580	−14.6200
	(0.4750)	(1.0410)	(0.5370)	(1.0400)	(1.0460)	(0.7800)	(1.1000)	(1393.8000)
3 days post DS	−0.1130	0.4850	−14.2000	−14.8200	−0.1550	−13.6600	0.7790	1.0410
	(0.6190)	(0.6250)	(685.2000)	(1993.4000)	(1.0840)	(658.9000)	(1.1420)	(1.0420)
Temperature	0.0373 *	0.0561 *	0.0142	0.0202	0.1090 *	−0.0033	−0.0061	0.1680 **
	(0.0183)	(0.0241)	(0.0276)	(0.0521)	(0.0434)	(0.0323)	(0.0371)	(0.0537)
NO_2_	0.0065	0.0298	−0.0310	−0.0545	0.0011	−0.0260	0.0401	0.0707
	(0.0155)	(0.0195)	(0.0249)	(0.0481)	(0.0351)	(0.0299)	(0.0303)	(0.0385)
O_3_	0.0036	−0.0076	0.0188 *	−0.0102	0.0131	0.0032	0.0053	−0.0014
	(0.0059)	(0.0078)	(0.0087)	(0.0180)	(0.0129)	(0.0104)	(0.0127)	(0.0154)
Trend	−0.0004	0.0024	−0.0047	0.0099	−0.0091	−0.0056	−0.0079	0.0240 **
	(0.0028)	(0.0036)	(0.0043)	(0.0083)	(0.0063)	(0.0050)	(0.0059)	(0.0074)
Spring	−0.1770	−0.1130	−0.2300	0.3930	0.1780	−0.1590	−0.7880	0.0707
	(0.1840)	(0.2370)	(0.2870)	(0.5250)	(0.4140)	(0.3300)	(0.4270)	(0.4370)
Autumn	−0.1810	−0.1310	−0.2370	−0.0077	0.0976	−0.1030	−0.4600	−0.2790
	(0.1520)	(0.1960)	(0.2340)	(0.4620)	(0.3330)	(0.2660)	(0.3420)	(0.3950)
Winter	−0.0237	0.0691	−0.0906	0.4730	0.8670	−0.5710	−0.4190	0.6410
	(0.2480)	(0.3200)	(0.3870)	(0.7300)	(0.5710)	(0.4540)	(0.5190)	(0.6440)
AR1	0.0100	−0.0139	0.0014	−0.0091	−0.0103	−0.0036	0.0008	−0.0175
AR2	0.0211	0.0361 *	0.0110	0.0010	0.0046	−0.0072	−0.0052	−0.0052
AR3	0.0053	0.0142	−0.0044	−0.0002	−0.0053	−0.0055	−0.0064	−0.0005
AR4	−0.0022	0.0048	−0.0028	−0.0105	−0.0216	0.0075	−0.0012	−0.0107
AR5	0.0043	−0.0086	−0.0247	0.0087	−0.0173	0.0008	0.0003	−0.0111
AR6	0.0177	0.0138	0.0152	0.0000	−0.0219	0.0190	−0.0010	−0.0007
AR7	0.0020	−0.0052	0.0157	−0.031	−0.0167	0.0095	−0.0247	0.0522 *
*N*	1820	1820	1820	1820	1820	1820	1820	1820

* *p* < 0.05, ** *p* < 0.01, *** *p* < 0.001. Reference group: no-event summer day. The notation AR(k) indicates an autoregressive model of order k.

**Table 6:** Poisson autoregressive analysis for dry AMD inpatient care.

	Total	Male	Female	51–60	61–70	71–80	≥81
Intercept	0.8220	0.5160	−0.4700	−2.4830	−0.3430	0.4220	−0.2650
	(0.6780)	(0.8360)	(1.1660)	(2.9490)	(1.6560)	(1.1250)	(1.0020)
Day of DS	−0.1440	−0.1430	−0.2050	−16.0500	0.1710	−0.2590	−0.4070
	(0.3900)	(0.4580)	(0.7380)	(2555.3000)	(0.9620)	(0.7230)	(0.5870)
1 day post DS	0.8930 *	0.8940 *	0.9890	−16.6800	1.0710	0.8790	0.9810 *
	(0.3740)	(0.4490)	(0.6750)	(4133.4000)	(1.1240)	(0.6420)	(0.4940)
2 days post DS	−0.4020	−0.2610	−0.6250	−17.1300	−16.7900	0.3270	−0.5910
	(0.5290)	(0.6120)	(1.0610)	(4192.0000)	(3202.2000)	(0.7520)	(0.7410)
3 days post DS	−0.7360	−0.9950	−0.3620	−16.8400	0.925	−15.6100	−0.7110
	(0.7620)	(1.0600)	(1.1090)	(4784.5000)	(1.1160)	(1216.0000)	(1.0490)
Temperature	0.0276	0.0377 *	0.0068	−0.0316	0.0029	0.0271	0.0321
	(0.0154)	(0.0190)	(0.0265)	(0.0659)	(0.0376)	(0.0260)	(0.0225)
NO_2_	0.0262 *	0.0411 **	−0.0005	0.0272	0.0140	0.0182	0.0349
	(0.0130)	(0.0158)	(0.0230)	(0.0591)	(0.0325)	(0.0218)	(0.0189)
O_3_	−0.0007	−0.0089	0.0143	0.0469 *	0.0001	0.0069	−0.0127
	(0.0052)	(0.0065)	(0.0089)	(0.0198)	(0.0134)	(0.0085)	(0.0078)
Trend	−0.0170 ***	−0.0188 ***	−0.0139 **	−0.0135	−0.0199 **	−0.0207 ***	−0.0146 ***
	(0.0025)	(0.0031)	(0.0043)	(0.0108)	(0.0063)	(0.0041)	(0.0037)
Spring	0.0716	0.1690	−0.1570	−0.7150	−0.2580	−0.2440	0.4240
	(0.1610)	(0.1940)	(0.2880)	(0.7000)	(0.4320)	(0.2670)	(0.2320)
Autumn	0.0002	0.0121	−0.0570	−0.5880	0.2300	−0.1450	−0.0068
	(0.1360)	(0.1670)	(0.2350)	(0.5770)	(0.3360)	(0.2160)	(0.2090)
Winter	−0.1400	−0.1600	−0.1580	−1.3010	−0.0507	−0.2920	−0.0607
	(0.2140)	(0.2620)	(0.3720)	(0.9530)	(0.5320)	(0.3540)	(0.3140)
AR1	0.0054	0.0228	−0.0041	−0.0152	−0.0122	0.0003	0.0010
AR2	0.0083	−0.0031	−0.0052	−0.0132	−0.0085	−0.0071	0.0065
AR3	−0.0001	0.0000	−0.0043	−0.0211	−0.0278	−0.0086	−0.0017
AR4	−0.0080	−0.0035	−0.0015	−0.0196	0.0165	−0.0002	−0.0078
AR5	−0.0055	−0.0053	−0.0053	−0.0118	0.0334	−0.0048	−0.0027
AR6	−0.0158	−0.0135	−0.0059	−0.0127	0.0114	−0.0065	−0.0088
AR7	0.0130	0.0055	0.0151	0.0225	0.0186	0.0057	0.0027
*N*	1820	1820	1820	1820	1820	1820	1820

* *p* < 0.05, ** *p* < 0.01, *** *p* < 0.001. Reference group: no-event summer day. The notation AR(k) indicates an autoregressive model of order k.

## Data Availability

Not available.
